# The role of language in mental health during the transition from primary to secondary education

**DOI:** 10.1177/17470218231158069

**Published:** 2023-03-22

**Authors:** Maria Barbara Jelen, Sarah Louise Griffiths, Laura Lucas, Jo Saul, Courtenay F Norbury

**Affiliations:** 1 Division of Psychology and Language Sciences, University College London, London, UK; 2 Department of Special Needs Education, University of Oslo, Oslo, Norway

**Keywords:** Language, mental health, language disorder, longitudinal

## Abstract

We report a preregistered analysis to test whether children meeting diagnostic criteria for language disorder (LD) have higher self-reported and/or parent-reported mental health symptoms during the transition from primary to secondary education. Data are from a UK-based longitudinal cohort study, The Surrey Communication and Language in Education Study (SCALES). SCALES oversampled children at risk of LD at school entry. Language was measured using a battery of standardised assessments in Year 1 (age 5–6 years, *n* = 529), and mental health symptoms were measured using self and parent report in Year 6 (age 10–11 years, *n* = 384) and Year 8 (age 12–13 years, *n* = 246). Social experiences were also measured using self-report measures in Year 6. Mental health symptoms were stable during the transition from primary to secondary school. Symptom rates did not differ between children with and without LD based on self-report, but children with LD had higher parent-reported mental health symptoms than their peers with typical language. Similarly, early language was negatively associated with parent-reported but not self-reported mental health symptoms. Early language was associated with fewer child-reported positive social experiences in Year 6, but social experiences did not mediate the association between language and mental health. We found poor agreement between parent and self-reported child mental health symptoms across language groups. Future studies should aim to determine sources of disagreement between parent and child report, particularly for children with communication difficulties who may struggle to accurately self-report mental health symptoms.

## Introduction

As a period of significant inter- and intrapersonal changes, early adolescence is a time of particular vulnerability to mental health difficulties. The onset of many mental health conditions begins in late childhood and adolescence (Kessler et al., 2005), which coincide with significant life events such as the transition to secondary school, which may, in turn, exacerbate stress (Coelho & Romão, 2016) and introduce significant changes to the social network and social support (Cantin & Boivin, 2004; Martínez et al., 2011).

The risk of mental health challenges during early/middle adolescence (11–15 years) is heightened for youth with speech-language disorders (Beitchman et al., 1996; Conti-Ramsden & Botting, 2008; St Clair et al., 2019), including Developmental Language Disorder (DLD). DLD is characterised by difficulties with learning and using language and affects approximately 7% of children (Norbury et al., 2017; Tomblin et al., 1997). Children with DLD are reported to be at increased risk of emotional, behavioural, and attentional-deficit/hyperactivity problems during adolescence (Conti-Ramsden & Botting, 2008; Yew & O’Kearney, 2013) relative to peers with typical language development. Behavioural and emotional difficulties may resolve by adulthood (Beitchman et al., 2001), but emotional problems remain higher in children with DLD during middle adolescence than for typically developing peers (St Clair et al., 2011). Social difficulties and poorer friendship quality, predicted by early language, are persistent challenges for children with language disorders (LD) and may contribute to poor mental health (Durkin & Conti-Ramsden, 2007).

The relationship between LD and mental health is likely to be multifactorial. Findings from the Twin Early Development Study (TEDS) identified strong genetic correlations between internalising symptoms (such as feelings low self-esteem, social withdrawal or anxiety/depression symptoms (Guzman-Holst & Bowes, 2021) and DLD, indicating that their co-occurrence may reflect common genetic influences (Toseeb et al., 2021). The presence of DLD may also exacerbate internalising symptoms due to the effect of language on identifying and managing emotional states (Griffiths et al., 2021) and building peer relationships.

Social relationships are facilitated by verbal interactions and may therefore be more difficult to navigate for adolescents with LD (St Clair et al., 2011). Early adolescence coincides with the transition to secondary education, and this has been associated with a decline in friendship numbers and changes to established social networks in the general adolescent population (Cantin & Boivin, 2004).

Social relationships are highly important for adolescent mental health. Lack of friends and/or social popularity has been associated with increased incidence of depressive symptoms and decreased self-worth in the general population (Rubin et al., 2006). Number of close friends directly predicts happiness in early teenage years (Uusitalo-Malmivaara & Lehto, 2013), and both children (Redmond, 2011) and adolescents (Durkin & Conti-Ramsden, 2007) with LD have been found to have lower quality friendships and fewer friends overall (Fujiki et al., 1996), compared with typically developing peers. Pupils with LD may be less liked by classmates (Andrés-Roqueta et al., 2016) and struggle with establishing social relationships due to difficulties with linguistic, social, and emotional processing. The peer relationships themselves may change, becoming more complex and expanding to encompass new, challenging types of pairings, such as romantic relationships or bully–victim interactions (Brown & Larson, 2009).

Young people with DLD are also at an increased risk of bullying (Conti-Ramsden & Botting, 2004; Hughes et al., 2013), due in part to communication difficulties and reduced experiences of the protective factor of friendship (Alvord & Grados, 2005). Bullying often leads to increased internalising symptoms (such as loneliness and withdrawal, as well as symptoms of depression or social anxiety), poor classroom attention, and suicidal ideation (Redmond, 2011), and bullying is a significant predictor of internalising symptoms in young people with DLD (Kilpatrick et al., 2019).

Not all children with DLD, however, have poor social outcomes. Mok and colleagues (2014) reported that 2/3 of their DLD sample exhibited childhood- or adolescent-onset peer difficulties, but 1/3 reported low or no social difficulties. The strongest predictor of positive outcome was prosocial behaviour (engaging in behaviour beneficial for others with not expectation of benefit for oneself), such as comforting or sharing (Over, 2018), often characterised by more advanced pragmatic language skills, which may be difficult for some children with LD (Fujiki et al., 1999). This is in line with prior research reporting that prosocial behaviour is negatively correlated with behavioural difficulties in middle childhood (Farmer, 2000). Prosocial behaviour may therefore be a “protective factor” against social difficulties (Mok et al., 2014), and thus, mental health issues, for children with DLD.

Inconsistent and nuanced findings regarding the relationship between language and mental health may be partially attributable to the variability in how outcomes of interest are measured. In a recent study of the relationship between child language and adolescent mental health, Thornton and colleagues (2021) investigated if vocabulary at age 5 predicts internalising symptoms in adolescence, with self-reported symptoms as a primary outcome measure and parent-reported symptoms as a secondary measure. They found better early vocabulary to predict *worse* self-reported adolescent internalising symptoms, but the effect was reversed in parent report, with better vocabulary predictive of better mental health. The authors suggest children with higher language ability in childhood may experience more pressure to succeed academically, and therefore have a higher risk of stress and mental health difficulties in adolescence (Thornton et al., 2021), which may not be picked up on by parents. A potential limitation was that the language variable was composed exclusively of a vocabulary measure, not allowing for confident extrapolation to other aspects of language.

This discrepancy between parent and child report is common in developmental research. Although both aim to assess one underlying construct (child mental health), there is evidence of frequent discrepancy between the two (De Los Reyes & Kazdin, 2005). Correlations between parent and child report vary between populations and studies, but typically range from low to modest; for example, an average correlation of *r* = .28 was reported in a meta-analysis of 341 studies utilising parent- and child-reported mental health (De Los Reyes et al., 2015), and between *r* = .17–.58 in a population study of 12,861 parent–adolescent dyads from 25 varied societies (Rescorla et al., 2013). Adolescents typically report more symptoms than caregivers (Petot et al., 2011; van der Ende & Verhulst, 2005), and possible explanations include adults’ tendency to report fewer symptoms overall, or to report more apparent externalising symptoms while underreporting internalising symptoms that the adolescent may not be explicitly sharing (Rescorla et al., 2013). The current study includes both child- and parent-reported mental health measures, and these will be considered separately in the main analyses.

In summary, some adolescents with LD appear to have poorer mental health outcomes compared with their peers, and it is not comprehensively understood why this is the case. Factors associated with the vulnerability to increased symptoms include social difficulties and experiences of bullying, while increased prosocial behaviour has been indicated as a potential protective factor against symptoms. It is therefore worth investigating if and how these constructs impact the mental health of individuals with LD across multiple points in development. Furthermore, the timing of the current study coincided with the COVID-19 pandemic, leading to an opportunity for exploratory investigation into its effects on mental health. Finally, due to a frequently observed discrepancy between child- and parent-reported scores of mental health symptoms, there is a debate regarding which measure is most accurate in describing adolescents’ experience; therefore, both measures were employed in this analysis. As well as direct causal pathways from language challenges to mental health symptoms, there are also a number of third factors that may explain the higher rates of mental health symptoms in children with LD compared with their peers. Sex, socioeconomic status (SES), and non-verbal IQ (NVIQ) have been independently associated with both LD and mental health outcomes. DLD has a slightly higher prevalence in boys compared with girls (Norbury et al., 2017; Tomblin et al., 1997); however, girls may experience higher levels of anxiety, depression, as well as peer-related and academic stress (Coelho & Romão, 2016; Evans et al., 2018), possibly leading to different patterns of mental health problems. Lower SES is directly associated with poorer language (McGillion et al., 2017; Pace et al., 2017), and it is an established risk factor for poor mental health (McLaughlin et al., 2012; McLeod & Shanahan, 1993). Many children with DLD also have low non-verbal IQ, although Norbury et al. (2016) found children with DLD and low-average NVIQ do not show markedly worse language or emotional and behavioural outcomes to their average-IQ counterparts. Children with lower non-verbal IQ are also at higher risk of being bullied (Verlinden et al., 2013), which could exacerbate the mental health difficulties of children with LD.

In this article, we report a preregistered analysis (https://osf.io/yg2wf) to test whether there is an increase in mental health symptoms between late childhood and early adolescence. We predicted that children with LD would have poorer mental health over this period and that social relationships and experiences would mediate the relationship between childhood language and early adolescent mental health. We have included the variables of sex, SES, and non-verbal IQ as control variables in the model to assess if they are directly related to the outcome variable (mental health) and to check for their moderation effects on key independent variables of interest. In addition, we report a preregistered exploratory analysis examining the effect of COVID-19-related lockdowns on adolescent mental health, as the school closures occurred during data collection and the pandemic has been found to have impacted on adolescent mental health (Barendse et al., 2021; Wright et al., 2021).

Our primary preregistered hypotheses were the following:

1. Mental health symptoms (anxiety and depression) will increase between Year 6 and Year 8. Greater symptom increase will be associated with lower language levels in Year 1.
2. Children with LD will experience increased symptoms of poor mental health compared with typically developing peers between Year 6 (age 10–11) and Year 8 (age 12–13).
3. Negative and positive social experiences will mediate the relationship between language and mental health symptoms in secondary school.
  - Language will be positively related to good social skills and peer relations, and these will mediate the relationship between language and mental health symptoms (good social/peer skills as protective factors)
  - Language will be negatively associated with bullying (i.e. children with LD will experience more bullying than peers without LD)—bullying will in turn increase risk for adverse mental health symptoms.

We additionally tested whether adolescents tested in Year 8 after Covid-19 lockdown had increased symptoms of anxiety and depression relative to peers tested in Year 8 prior to Covid-19 lockdown, so that we could control for any effect of lockdown in our analysis (note, this is a between-subjects comparison). Based on prior research, we hypothesised that children seen in lockdown would have poorer mental health than those seen prior to lockdown. However, our longitudinal study was not designed to investigate the effects of the pandemic and we do not have sufficient longitudinal data to draw firm conclusions regarding the effects of the pandemic on mental health symptoms of young people with LD.

## Methods

### Sampling

Participants for the current study were part of the Surrey Communication and Language in Education Study (SCALES) cohort, consisting of children who entered state-maintained education in Surrey in the United Kingdom, in September 2011. Upon entry, the children were screened for difficulties with language and communication using the Children’s Communication Checklist-Short; CCC-S (Norbury et al., 2017), a teacher-report questionnaire. Based on this assessment, the children were classified as having (1) no phrase speech (NPS—rated by teacher as unable to combine words into phrases or sentences) (2) high risk for DLD, and (3) low risk for DLD. Children were classified as high risk if they were scored in the top 14th centile on the CCC-S for their age and sex.

A total of 529 monolingual children (39 NPS, 200 low risk, 290 high risk) from the original cohort underwent further in-depth assessments in Year 1 (T2, age 5–6 years, *n* = 529), Year 3 (T3, age 7–8 years, *n* = 499), Year 6 (T4, age 10–11 years, *n* = 384), and Year 8 (T5, age 12–13 years, *n* = 246). Selection into the follow-up subset was determined using stratified random sampling, which oversampled children at risk for DLD (Norbury et al., 2016). Initial exclusion criteria included attending a special school and speaking English as a second language.

Attrition at T5 was affected by the Covid-19 pandemic; 197 children were seen at school prior to Covid-19 UK lockdown on 26 March 2020 (testing between September 2019 and March 2020), and 49 children were tested online during the Covid-19 lockdown period (between April 2020 and June 2020). Despite our efforts to test participants during the lockdown, the group tested during lockdown included markedly few responses from parents (Before: LD = 22, No LD = 88; During: LD = 4, No LD = 35) and children (Before: LD = 48, No LD = 139; During: LD = 2, No LD = 44) with LD.

### Ethical approval and consent

Consent procedures and study protocol for the SCALES study were developed in consultation with Surrey County Council and approved by the UCL Research Ethics Committee (9733/002). Informed consent was collected from parents at T2, prior to in-depth assessments. Consent from parents for the assessment of their children at T4 (Year 6) and T5 (Year 8) was obtained at T4. Eighty additional children, who were not assessed in T4, returned to the cohort at T5, and consent was obtained from parents for their participation at that time. Child assent was collected at T4, T5, and T6. Children were rewarded for their participation after each session with certificates and small prizes.

### Participants

#### Language

Testing at T2 included six language assessments: the Receptive One-Word Picture Vocabulary Test (ROWPVT-4; Martin & Brownell, 2011), Expressive One-Word Picture Vocabulary Test (EOWPVT-4; Martin & Brownell, 2011), School-Age Sentence Imitation Test (SASIT E32; Marinis et al., 2011), Assessment of Comprehension and Expression 6–11 Narrative Recall (ACE 6–11; Adams et al., 2001), Assessment of Comprehension and Expression 6–11 Narrative Comprehension (ACE 6–11; Adams et al., 2001), and the Test of Reception of Grammar—Short Form (TROG-S) (Bishop, 2003). Z-scores on for each of these measures were used to calculate six language composite scores for expressive and receptive vocabulary, grammar, and narrative skills (Norbury et al., 2017). A single overall language composite score was calculated by averaging the z-scores of all six language measures. The factor structure of the six language variables was previously modelled by Goh and colleagues (2021) using confirmatory factor analysis. They found that a single-factor language model was a best fit to the data, supporting the current study’s operationalisation of language using a composite score.

#### T2 language disorder category

A child was categorised as having LD if they scored −1.5 *SD* on at least 2 out of 6 of the composite scores in Year 1. The LD group included children with no known associated biomedical condition (Year 1 *N* = 91, Year 3 *N* = 87) and those with a comorbid condition such as intellectual disability (a score of −2 *SD* or greater on a composite non-verbal IQ score) or an existing diagnosis, for example, autism (Year 1 *N* = 45, Year 2 N = 42; Norbury et al., 2017).

#### Non-verbal IQ

Non-verbal IQ was measured at T2 using a composite of the Wechsler Preschool and Primary Scale of Intelligence (WPPSI-III; Wechsler, 2003) Block Design and Matrix Reasoning subscales.

#### Mental health outcomes

Anxiety and depression were measured using the 25-item Revised Children’s Anxiety and Depression Scale (RCADS-25; Ebesutani et al., 2012). The child self-report short version was the primary outcome measure, while the parent-report short version (RCADS-25-P) was a secondary measure. Depression and anxiety subscale scores were analysed separately. The child and parent rated their how often they/their child experience statements relating to anxiety and depression on a scale from 0 to 4 (“never,” “sometimes,” “often,” “always”). RCADS is reported to have good reliability in both clinical (Chorpita et al., 2005) and school-based child and adolescent samples (Piqueras et al., 2017).

#### Social experiences

Social experiences were measured using The Life in School Checklist (Arora, 1994) and two subscales from the Strengths and Difficulties Questionnaire (SDQ) (Goodman, 1997). We used 16 items from the original 40-item Life in School Checklist, including 8 items tapping positive interactions with peers (e.g., “was very nice to me”) and 8 items tapping negative interactions with peers (e.g., “told a lie about me”). Children indicated the frequency of each type of interaction within the past week and total positive/ negative experiences score out of 8 was calculated. In addition, we used the prosocial subscale of the SDQ as a measure of positive social experiences and the peer problems subscale of the SDQ to measure negative social experiences. Participants rated behaviours and attitudes on a 3-point, Likert-type scale (“not true,” “somewhat true,” “certainly true”). A total score between 0 and 10 was calculated for each subscale, with higher scores indicative of greater prosocial behaviour/peer problems. In the analysis, subscale scores from both measures were used to create two latent variables: one for positive and one for negative social experiences.

#### Socioeconomic status

The Income Deprivation Affecting Children Index (IDACI) was used as a measure of SES (McLennan et al., 2011). The index ranks individual neighbourhoods in England on the basis of local employment and receipt of benefits, with rank scores ranging from 1 (most deprived) to 32,844 (least deprived). The scores in our sample ranged from 3908 to 32,471, with a mean rank of 21,351 (*SD* = 7755).

### Statistical analyses

All analyses were carried out using R version. 4.1.0. (R Core Team, 2017) in R Studio version 1.2.5001 (RStudio Team, 2020), using the packages lme4 version 1.1.27. (Bates et al., 2015) and lmerTest version 3.1.3. (Kuznetsova et al., 2017) for Linear Mixed Effects (LME) models, the ez version 4.4-0 (Lawrence, 2011) for ANOVA and lavaan v. 0.6-9. (Rosseel, 2012) for Structural Equation Modelling (SEM).

#### Hypotheses 1. Mental health symptoms (anxiety and depression) will increase between Year 6 and Year 8. Greater symptom increase will be associated with lower language levels in Year 1

First, to establish whether there is a change in mental health between Year 6 and Year 8, Linear Mixed Effect (LME) models with a random effect of participant and fixed effects of time (Year 6 and Year 8 measurements), language score, age within school year (z-score giving age relative to class peers), sex, SES, and NVIQ were created for each outcome measure. We also included a Covid-19 variable as a fixed effect that indicated whether the child had been seen in Year 8 before or after the Covid-19 lockdown to establish whether this effected mental health outcomes in our sample. We included the interaction between time and language in Year 1, time and lockdown, and time and sex. The interaction between time and lockdown was included because we expected that children seen during lockdown in T5 would show a greater increase in mental health symptoms between T4 and T5, compared with those seen prior to lockdown in T5. Separate models were constructed with each of the four outcome variables: self-reported depression, self-reported anxiety, parent-reported depression, and parent-reported anxiety.

#### Hypothesis 2. Children with LD will experience increased symptoms of poor mental health compared with typically developing peers between Year 6 (age 10–11) and Year 8 (age 12–13)

A 2 (group) × 2 (time) ANOVA was used to assess the hypothesis that children with LD have poorer mental health in Year 6 and a greater increase in symptom severity between Year 6 and Year 8 relative to peers. We compared the group scores across the two time points, with the interaction between time and group testing for a difference in rates of change between the two groups.

#### Hypothesis 3. Negative and positive social experiences will mediate the relationship between language and mental health symptoms in secondary school

**Figure 1.**
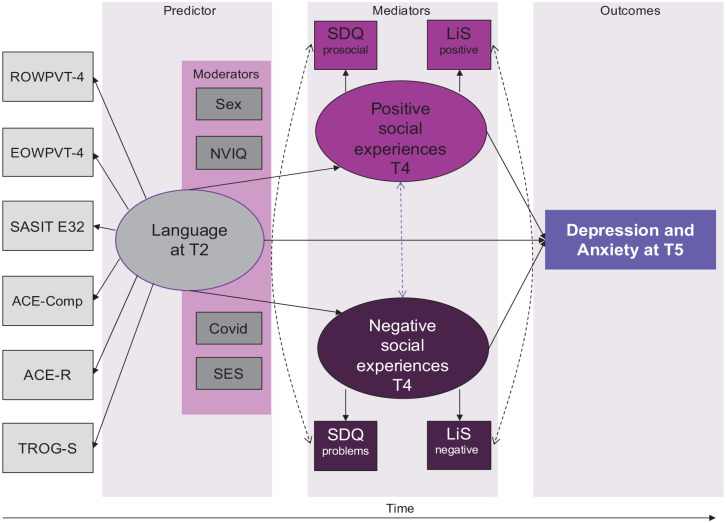
Diagrammatic representation of SEM analysis model.

We used SEM with mental health scores (four separate models for parent versus child rated anxiety and depression, see Figure 1) in Year 8 as the outcome variable, a latent language variable in Year 1 as a predictor and two latent variable mediators: positive social experiences (measured by Positive Interactions and Prosocial Behaviours) and negative social experiences (Bullying and Peer Difficulties). Participant sex, SES, experience of the Covid-19 lockdown, and non-verbal intelligence were included as moderators.

Model fit was assessed using the chi-square test, the root-mean-square error of approximation (RMSEA; acceptable fit: <.08, good fit: <.05), the comparative fit index (CFI; acceptable fit: .95–.97, good fit: >.97), and the standardised root-mean-square residual (SRMR; acceptable fit: .05–.10, good fit: <.05; Schermelleh-Engel et al., 2003).

To account for missing data, we used the full information maximum likelihood (FIML) data estimation approach, which allows for unbiased parameter estimates and standard errors (Enders & Bandalos, 2001). Under this approach, a likelihood function for each individual is estimated based on existing variables, so as to maximise the use of available data.

## Results

### Descriptive statistics

The final analysis included demographic and language data from 529 children (LD = 137) in Year 1; 350 children (LD = 80) and 185 parents (LD = 31) in Year 6 and 233 children (LD = 50) and 150 parents (LD = 26) in Year 8. The LD and typical language groups did not differ on age, but children with LD had, on average, significantly lower SES and NVIQ scores (Table 1).

**Table 1.** Characteristics of children with language disorder versus typical language development.

		LD		No LD	
Sex distribution in Year 6 (*N* / % female)		36 / 45.0%		141 / 52.2%	
Sex distribution in Year 8 (*N* / % female)		21 / 42.0%		95 / 51.9%	
Mean			*SD*	Mean	*SD*
Age in Year 6 (months)		133.53	4.27	133.96	4.24
Age in Year 8 (months)^a^		152.76	4.21	154.39	4.92
Language (z-scores)^a^		−2.07	0.69	−0.12	0.81
SES (IDACI rank)^a^		18290	7717	22428	7485
NVIQ (z-scores)^a^		−1.19	1.05	−0.16	0.97
Positive social experiences	LiS positive^a^	18.00	5.50	20.538	2.929
	SDQ Prosocial Behaviour	7.22	2.68	8.04	1.75
Negative social experiences	LiS negative^a^	10.44	3.01	11.08	4.20
	SDQ Peer Difficulties	2.33	1.32	2.10	1.98
Depression in Year 6	Child-report	8.36	5.77	7.29	4.97
	Parent-report	4.90	3.68	3.04	3.77
Depression in Year 8	Child-report	8.50	5.21	8.16	4.71
	Parent-report	6.92	5.97	4.06	3.95
Anxiety in Year 6	Child-report	11.60	8.31	10.66	7.43
	Parent-report	8.77	5.77	4.74	5.04
Anxiety in Year 8	Child-report	11.24	8.35	11.01	6.96
	Parent-report	9.50	8.38	6.12	5.00

LD: language disorder; *SD*: standard deviation; SES: socioeconomic status; IDACI: Income Deprivation Affecting Children Index; NVIQ: non-verbal IQ; SDQ: Strengths and Difficulties Questionnaire.

a Mean values for children with LD and without LD differed significantly in the case of age in Year 8, *t*(91) = 2.20, *p* = .03, language, *t*(279) = 27.23, *p* < .001, SES, *t*(234) = 5.46, *p* < .001, NVIQ, t(223) = 10.02, *p* < .001, as well as both the positive, t(118) = 2.71, *p* = .008, and negative, t(132) = –3.57, *p* < .001, subscales of the Life in Schools checklist.

### Hypothesis 1. Mental health symptoms will increase between Year 6 and Year 8 and lower language will be associated with greater increase

**Figure 2.**
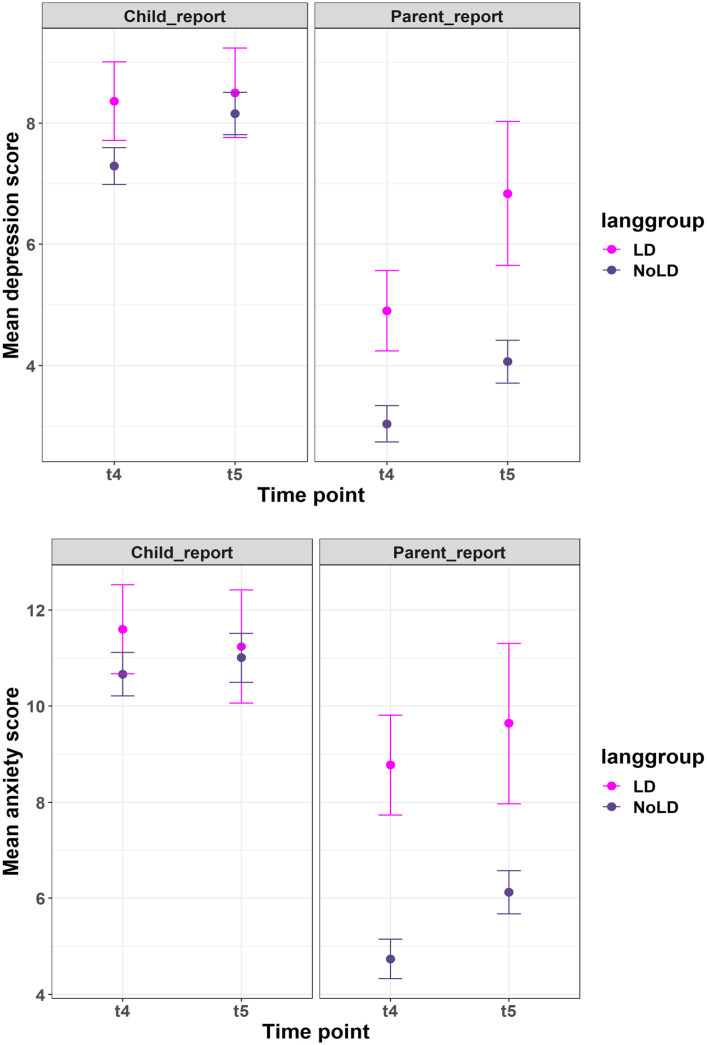
Anxiety (top) and depression (bottom) scores in Year 6 and Year 8, for participants with language disorder versus typical language development, parent and child report. Maximum RCADS-25 score was 75, where a score of 65 or above indicates borderline or clinical threshold.

Mean symptoms of anxiety and depression in each language group at each time-point are presented in Figure 2. The results of the LME models are presented in Tables 2 and 3. There was no significant effect of time in any of the LME models indicating that symptoms did not increase between Year 6 and Year 8. For child-reported symptoms, language was not a significant predictor of mental health in either Year 6 or Year 8. For parent-reported anxiety and depression, better language was associated with fewer parent-reported symptoms both in Year 6 and Year 8. However, there was no significant interaction between language and time, indicating that language in Year 1 was not associated with a greater increase in symptoms between Year 6 and Year 8.

**Table 2.** Unstandardised estimates, 95% confidence intervals and *p* values from of LME models of child-reported depression and anxiety. Significant *p* values are in bold font.

**Predictors**	**Child report**					
	Depression			Anxiety		
	Estimates	CI	*p*	Estimates	CI	*p*
**(Intercept)**	9.12	6.99 to 11.26	<.001	11.35	8.30 to 14.40	<.001
**Time [T5]**	−0.03	−1.09 to 1.03	.960	−0.29	−1.86 to 1.28	.719
**Language**	−0.31	−1.02 to 0.39	.384	−0.40	−1.42 to 0.62	.441
**Sex**	−0.05	−1.43 to 1.34	.947	2.54	0.55 to 4.53	**.013**
**SES**	0	−0.00 to 0.00	.093	0	−0.00 to 0.00	.228
**NVIQ**	−0.02	−0.72 to 0.68	.955	0.02	−0.97 to 1.01	.975
**Lockdown**	−0.17	−1.94 to 1.60	.850	−0.69	−3.23 to 1.86	.597
**Age**	−0.10	−0.66 to 0.45	.723	−0.25	−1.05 to 0.55	.540
**Time [T5] × Language**	0.09	−0.51 to 0.70	.759	−0.06	−0.96 to 0.84	.895
**Time [T5] × Sex**	1.57	0.24 to 2.89	**.021**	1.00	−0.96 to 2.96	.321
**Time [T5] × Lockdown**	−0.81	−2.61 to 0.99	.379	−1.33	−4.00 to 1.33	.328

CI: confidence interval; SES: socioeconomic status; NVIQ: non-verbal IQ.

**Table 3.** Unstandardised estimates, 95% confidence intervals, and *p* values from of LME models of parent-reported depression and anxiety. Significant *p* values are in bold font.

**Predictors**	**Parent report**					
	Depression			Anxiety		
	Estimates	CI	*p*	Estimates	CI	*p*
**(Intercept)**	6.35	3.98 to 8.71	<.001	9.67	6.40 to 12.95	<.001
**Time [T5]**	0.29	−0.71 to 1.30	.572	0.36	−0.77 to 1.49	.535
**Language**	−0.68	−1.42 to 0.07	.077	−1.04	−2.05 to −.03	**.046**
**Sex**	−0.52	−1.97 to 0.93	.486	0.04	−1.89 to 1.97	.968
**SES**	0	−0.00 to −.00	**.032**	0	−0.00 to −.00	**.021**
**NVIQ**	−0.21	−0.95 to 0.52	.570	−0.34	−1.36 to 0.68	.513
**Lockdown**	−0.69	−2.27 to 0.89	.395	−1.35	−3.49 to 0.78	.216
**Age**	0.19	−0.38 to 0.75	.515	0.19	−0.51 to 0.89	.602
**Time [T5] × Language**	−0.09	−0.62 to 0.43	.727	0.01	−0.57 to 0.59	.974
**Time [T5] × Sex**	0.38	−0.83 to 1.60	.536	0.66	−0.69 to 2.01	.340
**Time [T5] × Lockdown**	0.4	−1.04 to 1.85	.584	−0.4	−2.03 to 1.22	.627

CI: confidence interval; SES: socioeconomic status; NVIQ: non-verbal IQ.

There was a main effect of sex on child-reported anxiety with girls reporting greater anxiety (Year 6: *M* = 12.19, *SD* = 7.80; Year 8: *M* = 12.66, *SD* = 7.59) than boys (Year 6: *M* = 9.53, *SD* = 7.24; Year 8: *M* = 9.47, *SD* = 6.58). There was also an interaction between sex and time on child-reported depression that resulted from increasing depression from Year 6 to Year 8 for girls only (Year 6: *M* = 7.63, *SD* = 5.20; Year 8: *M* = 8.90, *SD* = 5.02). No other main effects or interactions were significant in child-reported mental health scores.

In parent-report data, language in Year 1 was a significant predictor of anxiety scores in Year 8 and was marginally associated with depression ratings in Year 8. There was an additional effect of SES, with parents from less affluent backgrounds reporting more symptoms than those from more affluent families.

### Hypothesis 2. Children with LD will experience increased symptoms of poor mental health compared with typically developing peers between Year 6 (age 10–11) and Year 8 (age 12–13)

The ANOVA models with LD group as a predictor of mental health symptoms echoed the findings of the LME models. There was no effect of time in any of the models. There was a significant effect of language group for parent-reported, but not child-reported anxiety and depression symptoms, with children with LD experiencing more symptoms in both Year 6 and Year 8 relative to children with typical language. However, the interaction between time and language group was not significant, showing children with LD do not experience a greater increase in symptoms between Year 6 and Year 8 (Table 4).

**Table 4.** ANOVA comparing symptom change between Year 6 and Year 8, for LD and No LD groups.

		Predictor	DFn	DFd	*F*	*p*	ges
**Anxiety**	Child	Time	1	439	0.04	.85	0.00
		Language group	1	439	0.27	.60	0.00
		Time × Language group	1	439	0.08	.78	0.00
	Parent	Time	1	261	2.53	.11	0.01
		**Language group**	**1**	**261**	**20.04**	**.00**	**0.07**
		Time × Language group	1	261	0.21	.65	0.00
**Depression**	Child	Time	1	439	0.82	.36	0.00
		Language group	1	439	1.25	.26	0.00
		Time × Language group	1	439	0.37	.54	0.00
	Parent	Time	1	262	3.34	.07	0.01
		**Language group**	**1**	**262**	**14.70**	**.00**	**0.05**
		Time × Language group	1	262	0.03	.87	0.10

DFn, DFd: degrees of freedom; ges: generalized eta squared, an effect size.

Bold indicate significant paths at *p* < .05.

### Hypothesis 3. Negative and positive social experiences will mediate the relationship between language and mental health symptoms in secondary school

We next assessed whether the relationship observed between language and mental health in Year 8 could be explained by children’s positive and negative social experiences in Year 6. Means and standard deviations for each measure of social experiences for each language group are given in Table 1. Children with LD reported significantly fewer positive experiences, *t*(118) = 2.71, *p* = .008, and fewer negative experiences, *t*(132) = –3.57, *p* < .001, on the Life in Schools Checklist, compared with children without LD. They also reported slightly less prosocial behaviour, *t*(120) = 1.74, *p* = .09, and exhibited more peer difficulties, *t*(143) = –1.76, *p* = .08, but these differences did not reach conventional levels of statistical significance.

**Figure 3.**
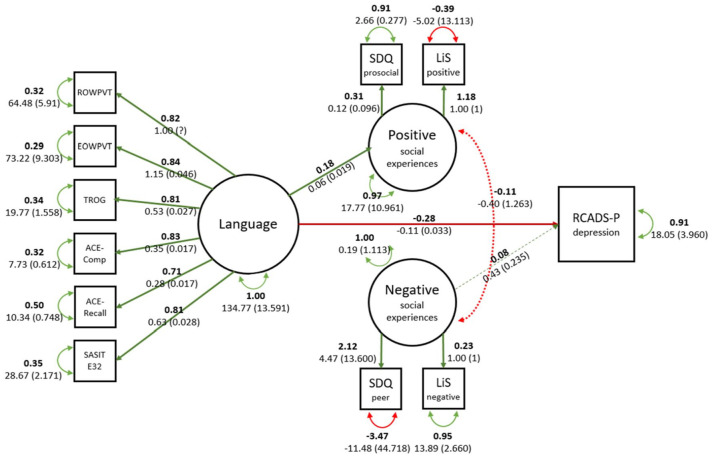
Visual representation of parent-reported depression SEM model.

**Figure 4.**
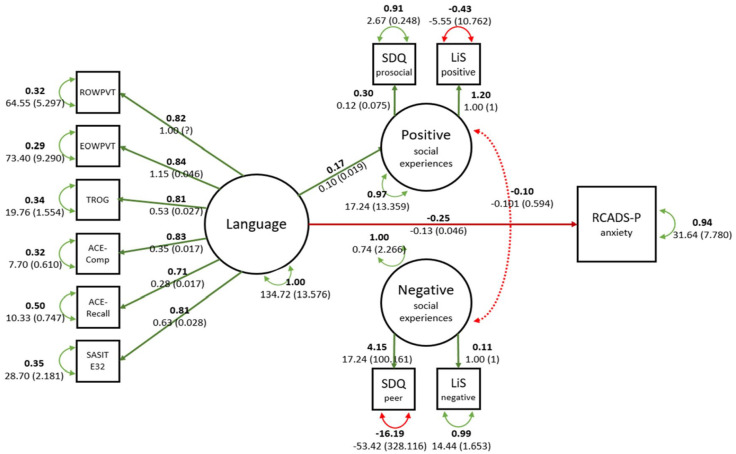
Visual representation of parent-reported anxiety SEM model.

Year 1 language did not predict child-reported Year 8 mental health symptoms, so it was not possible to test mediation for the child report data. We therefore report mediation models for parent-report outcomes only. The fit of the parent-reported SEM depression model was acceptable (χ^2^ = 129.067, RMSEA = 0.065, CFI = 0.957, SRMR = 0.057) as was the fit for the anxiety model (χ^2^ = 125.176, RMSEA = 0.066, CFI = 0.957, SRMR = 0.060). Models and parameter estimates are given in Figures 3 and 4. Paths from Year 1 language to parent-reported mental health symptoms Year 8 were significant as were paths from language to Year 6 positive social experiences. Child-reported positive and negative social experiences were weakly related to depression, but there was no mediation effect of social experiences on the relationship between language and mental health in either model.

### Exploratory analysis: parent and child agreement

The correlations between child- and parent-reported scores in Year 6 and Year 8 were low to moderate (depression in Y6, *r*(168) = .24, *p* = .002, depression in Y8, *r*(141) = .41, *p* < .001, anxiety in Y6, *r*(166) = .24, *p* = .002, anxiety in Y8, *r*(141) = 0.38, *p* < .001). Child-report means were almost twice as high as those of parents, and there was poor agreement and consistency between parent and child scores (Table 5). Intraclass correlation coefficients (ICC) between parents and children report in the LD group were higher than in the typical language group.

**Table 5.** ICC (absolute agreement and consistency) illustrating inter-rater reliability between child and parent reporters (higher consistency in each LD/No LD group pair in bold).

		**Year 6**		**Year 8**	
		Agreement	Consistency	Agreement	Consistency
**Depression**	LD	**0.234**	**0.272**	**0.728**	**0.730**
	No LD	0.154	0.212	0.230	0.324
**Anxiety**	LD	**0.306**	**0.311**	**0.608**	**0.601**
	No LD	0.154	0.211	0.223	0.287

LD: language disorder.

### Exploratory analysis: role of Covid-19 lockdown

Testing in Year 8 was interrupted with the Covid-19 lockdown, resulting in some children being assessed before and some during the lockdown. Based on emerging findings evidencing the negative effects of the lockdown on child mental health, we included a cross-sectional exploratory analysis, comparing children measured in Year 8 before the lockdown to children measured in Year 8 during lockdown. The LME model found no interaction between time and lockdown (Tables 2 and 3), indicating children seen during lockdown in T5 did not have greater self- or parent-reported anxiety or depression scores compared with those seen prior to lockdown. See Table S2 in the online Supplementary Material for means and standard deviations of metal health scores of children seen before and after lockdown in Year 8.

## Discussion

We conducted a preregistered analysis to investigate self-reported and parent-reported mental health in children with and without LD during the transition from primary to secondary school. Based on previous research (Coelho & Romão, 2016), we predicted that there would be an increase in mental health symptoms between the last year of primary education and the second year of secondary education. Contrary to this hypothesis, we did not find any evidence for an increase in either parent- or child-reported mental health symptoms. We also predicted that children that met the criteria for LD in early primary school would experience poorer mental health in early adolescence, and that greater language ability in early primary school would predict fewer mental health symptoms. Here our findings differed depending on who was asked to report on a child’s mental health. Based on child self-report of mental health symptoms, we found no evidence of a difference between children with and without LD, and early language did not predict mental health symptoms. However, based on parent-report of mental health symptoms, children with LD did have higher anxiety and depression symptoms, and language ability in the start of primary school significantly predicted these mental health symptoms.

The difference in results between parent and child-report mental health is consistent with another recent study looking at the longitudinal association between language and mental health. Thornton et al. (2021) found poorer vocabulary measured at age 5 predicted higher parent-reported mental health symptoms at age 14. However, the *opposite* trend was found for child-reported mental health symptoms, with better vocabulary predicting worse mental health. The authors suggested this may reflect increased academic pressure for children with better language, of which parents are not aware. Alternatively, this could also indicate that adolescents with higher levels of vocabulary were able to better understand the questions in the self-reported measure and/or showed higher awareness of their mental state and therefore provide more accurate assessments of their symptoms. However, this assumption is very difficult to disentangle as the RCADS used in the current study is not accompanied by a measure of comprehension of the questionnaire. Accuracy is also challenging to assess, but if assumed to be a measure of agreement with parent-reported symptoms, our data did not support the claim that adolescents with better vocabulary are more accurate—higher agreement was observed for parents and children with LD. Furthermore, a straightforward association between better vocabulary and more symptoms cannot be drawn, as there is a decline in mental health symptoms in early adulthood compared with adolescence while vocabulary continues to increase.

When comparing child -parent agreement in children with and without LD, our findings closely match a recent study by Forrest et al. (2021). In a smaller sample, they also found significant group differences in parent, but not child-reported mental health symptoms, indicating more symptoms in children with language difficulties. These authors suggest the parents of children with LD may be biased by their perception of their children’s disorder leading them to overestimate their child’s perceived emotional difficulties.

Some previous studies with older adolescents who had received a formal DLD diagnosis have found increases in self-reported mental health symptoms (Conti-Ramsden & Botting, 2008; van den Bedem et al., 2018). For example, In the Manchester Language Study (Conti-Ramsden & Botting, 1999; Conti-Ramsden et al., 1997), mean symptoms of anxiety and depression reported by children with LD at age 16 were higher than those of typical peers (Conti-Ramsden & Botting, 2008). It may be that the younger age of the participants in our study meant they had more difficulty reporting mental health symptoms or that heightened mental health symptoms in children with LD do not occur until later in adolescence as they face increasing social and academic challenges as a result of LD. Another possibility is that children in these previous studies have more severe LD and/or more severe comorbid difficulties than children with LD in SCALES, because they were recruited from specialist language units, rather than from the mainstream school population. However, other studies of older adolescents with formal diagnoses have also not found differences in self-reported mental health symptoms, in line with our findings (Forrest et al., 2021; Kilpatrick et al., 2019).

The difference in results between parent and child report measures raises the question of which measure most accurately reflects child mental health status. Many authors suggest that self-report should be the primary measure for adolescent mental health, as parents may not be aware of internalising symptoms unless the young person chooses to disclose them (Thornton et al., 2021). In our study, which focused exclusively on internalising symptoms, child-reported symptoms were on average twice as high as reported by parents, a trend which has been reported in previous population studies (Rescorla et al., 2013), and fits with the idea that parents are underreporting internalising symptoms. It is also possible that differences between children and parents arise due to differences in understanding of the question items. Although the RCADS-25 (Ebesutani et al., 2012) asks parents and children identical questions, it is possible some items were interpreted differently by the child and the parent responders. A good example is the item, “I think about death,” which parents may interpret as a question about suicidal thoughts, whereas children may view it as being about the general concept of death, for example, in relation to the death of a book or video game character. Children may also be interpreting some questions more literally than parents, especially with regard to psychosomatic symptoms. As a result, children may report, for example, frequent tiredness but with regard to feeling tired after a busy day at school, and not necessarily a sense of exhaustion and apathy indicative of mental health problems.

Although we found low agreement between parents and children in both groups, as is typical in this age group (Rescorla et al., 2013), we actually observed *higher* agreement between children with LD and their parents. Greater agreement between parents and children with LD compared with parents and children with typical language may be due to increased time and attention that parents pay children with developmental disorders or special education needs, which may lead to better communication between the parent and child. Children with special educational needs (SEN) may also have more exposure to therapy or training on mental health awareness, increasing opportunities and capabilities for the child to share mental health concerns with parents. If this is the case, higher parent-reported mental health symptoms are a result of this increased attention to, rather than a true increase in symptoms.

Further research is needed to determine whether the parent and child RCADS is a valid tool for measurement of mental health in children with LD. The RCADS was initially validated for use with typically developing children, and showed adequate reliability and validity for children with attention-deficit/hyperactivity disorder (ADHD; Becker et al., 2019) and autism (Sterling et al., 2015), but has not been examined in samples including children with language difficulties, such as DLD. Establishing the utility of the RCADS and other mental health measures for this subpopulation is crucial for comprehensive understanding of their mental health. The identification of potential comprehension issues in questionnaires not designed with children with LD in mind may lead to the development and use of instruments specifically fine-tuned to the symptomology, and response ability, of the target respondents, as has been done, for example, for children with autism (Rodgers et al., 2016).

For parent-reported mental health symptoms, we were able to test whether quality of social relationships mediates the association between language and mental health symptoms (Forrest et al., 2021). Better language at school entry predicted more child-reported positive social experiences but did not predict negative social experiences. Child-reported negative social experiences predicted parent-reported depression. This is congruent with other studies highlighting the role of bullying and negative interactions in poor mental health (Kilpatrick et al., 2019; Redmond, 2011). However, contrary to our hypothesis, we did not find that negative social experiences mediated the relationship between language and mental health due to the lack of association between language and negative symptoms. The relationship between language and social experiences was also reflected in comparisons between children with LD and without LD. Children with LD reported fewer positive and *fewer* negative social experiences compared with peers. A lower number of both positive and negative experiences may indicate fewer friendships and social interactions of any valence in children with LD, as previously noted (Andrés-Roqueta et al., 2016; Durkin & Conti-Ramsden, 2007; Redmond, 2011).

Covid-19 school closures severely disrupted our final wave of data collection. Our exploratory analysis did not find evidence of poorer mental health for those seen during lockdown relative to those seen before pandemic onset. However, we note the relatively small number of children seen during lockdown and the difficulties of testing children with LD online, resulting in low statistical power to detect differences. This limits the conclusions we can draw by comparing mental health of these two groups of children. Some prior studies with a prime focus on COVID-19 have found that the experience of the pandemic was associated with a significant increase in depression symptoms in adolescents (Barendse et al., 2021; Wright et al., 2021); others, however, did not generally find a worsening in symptoms (Knowles et al., 2022). Although there is currently no research about the effect of the lockdown on children with DLD, heightened negative effects on functioning and mental well-being have been observed for children with autism (Colizzi et al., 2020) and ADHD (Bobo et al., 2020), suggesting a possible differential effect on children with developmental disorders. Although the current study was not designed to investigate this, the effects of the COVID-19 pandemic on the mental health of adolescents with LD would certainly be a valuable undertaking for future studies.

Strengths of the study include a comprehensive measurement of language using six standardised assessments of both receptive and expressive language skills. The longitudinal design and targeted sampling strategy of SCALES also allows for the study of developmental trajectories from childhood to adolescence, in well-matched groups of children with and without LD. The study also has limitations, notably the high rate of attrition into secondary school due to the Covid-19 pandemic, and the generally poorer rate of parent report, which may have exacerbated discrepancies between the child and parent responders. Furthermore, although the language composite used in the study was demonstrated to accurately represent a single-factor latent language variable (Goh et al., 2021), it crucially did not include a measurement of pragmatic language. As pragmatic language has social salience, it is possible pragmatic language skills independently contribute to how children are perceived and treated by peers. However, as a pragmatic language measure was not included in the initial language battery administered when participants were in Year 1, the current study was not able to assess if it is related to social or mental health difficulties. As discussed previously, the RCADS may also have limitations with regard to varying language abilities. Social experiences may also be difficult for children to assess—children may underreport peer difficulties or negative interactions because of unwillingness to report bullying to adults or lack of insight concerning peer interactions and perceptions.

In sum, our study paints a complex picture of the longitudinal relationships between LD and mental health during the transition to secondary school. Parent ratings suggest that children with LD have increased symptoms of anxiety and depression compared with their peers, and that poorer language in early primary school predicts more symptoms in early adolescence. In contrast, although all children reported higher symptom counts than their parents, the adolescents with LD do not report increased symptoms relative to peers with typical language. This lack of agreement between parents and children is puzzling and may be attributable to children understanding the questions differently to parents, but further investigation is warranted to determine the cause of the disagreement. The use of mental health measures tailored to the presentation and response capabilities of the children with LD may allow more reliable insight into their mental health, as achieved, for example, in applying a specialised scale for children with autism (Rodgers et al., 2016).

## Supplemental Material

sj-docx-1-qjp-10.1177_17470218231158069 – Supplemental material for The role of language in mental health during the transition from primary to secondary education Supplemental material, sj-docx-1-qjp-10.1177_17470218231158069 for The role of language in mental health during the transition from primary to secondary education by Maria Barbara Jelen, Sarah Louise Griffiths, Laura Lucas, Jo Saul and Courtenay F Norbury in Quarterly Journal of Experimental Psychology
