# Supplementary material for: The role of language in mental health during the transition from primary to secondary education
Source: Q J Exp Psychol (Hove). 2023 Mar 22;76(12):2732–48. doi: 10.1177/17470218231158069 (PMC10845840; doi:10.1177/17470218231158069)

# Supplementary Materials for:

The role of language in mental health during the transition from primary to secondary education

Jelen^1^, M.B, Griffiths^1^, S.L., Lucas^1^, L., Saul^1^, J. Norbury^1,2^, C.F.

1 Division of Psychology and Language Sciences, University College London

2 Department of Special Needs Education, University of Oslo

Address for correspondence:

Professor Courtenay Norbury

Psychology and Language Sciences

Chandler House

2 Wakefield Street

London

WC1N 1PF

Email: c.norbury@ucl.ac.uk

**Table S1.**

*Mean depression and anxiety scores before and during Covid-19 lockdown, child and parent report.*

|  |  | Before lockdown | | During lockdown | |
| --- | --- | --- | --- | --- | --- |
|  |  | Mean | SD | Mean | SD |
| Depression | Child | 8.50 | 4.99 | 7.13 | 3.91 |
|  | Parent | 4.75 | 4.69 | 3.97 | 3.86 |
| Anxiety | Child | 11.55 | 7.61 | 9.07 | 5.27 |
|  | Parent | 7.35 | 6.43 | 4.74 | 2.95 |

**Table S2.**

*Language and SES, split by assessment before and during lockdown*

|  |  | Before lockdown | | During lockdown | |
| --- | --- | --- | --- | --- | --- |
|  |  | Mean | SD | Mean | SD |
| Child-report | Language | -0.550 | 1.119 | 0.264 | 7529.357 |
|  | SES | 21462 | 7529 | 24915 | 6550 |
| Parent-report | Language | -0.314 | 1.254 | 0.057 | 7574.651 |
|  | SES | 22003.710 | 1.197 | 25028.900 | 6156.435 |

**Table S3.** *Correlation matrix for SEM models*

|  | rowpvttotal2 | eowpvttotal2 | trogtotal | narrativerecalltotal | narrativecomptotal | sentrepsentencetotal | LiSnegative_ct4 | LiSpositive_ct4 | SDQpeer_ct4 | SDQprosocial_ct4 | RCADS_dep_ct4 | RCADS_anx_ct4 | RCADS_dep_ct5 | RCADS_anx_ct5 | RCADS_dep_pt4 | RCADS_anx_pt4 | RCADS_dep_pt5 | RCADS_anx_pt5 | Sex | SES | lang | lockdown |
| --- | --- | --- | --- | --- | --- | --- | --- | --- | --- | --- | --- | --- | --- | --- | --- | --- | --- | --- | --- | --- | --- | --- |
| rowpvttotal2 |  |  |  |  |  |  |  |  |  |  |  |  |  |  |  |  |  |  |  |  |  |  |
| eowpvttotal2 | 0.72 |  |  |  |  |  |  |  |  |  |  |  |  |  |  |  |  |  |  |  |  |  |
| trogtotal | 0.66 | 0.64 |  |  |  |  |  |  |  |  |  |  |  |  |  |  |  |  |  |  |  |  |
| narrativerecalltotal | 0.5 | 0.5 | 0.56 |  |  |  |  |  |  |  |  |  |  |  |  |  |  |  |  |  |  |  |
| narrativecomptotal | 0.6 | 0.68 | 0.6 | 0.6 |  |  |  |  |  |  |  |  |  |  |  |  |  |  |  |  |  |  |
| sentrepsentencetotal | 0.63 | 0.63 | 0.66 | 0.5 | 0.6 |  |  |  |  |  |  |  |  |  |  |  |  |  |  |  |  |  |
| LiSnegative_ct4 | -0.2 | -0.14 | -0.2 | -0.1 | -0.2 | -0 |  |  |  |  |  |  |  |  |  |  |  |  |  |  |  |  |
| LiSpositive_ct4 | 0.18 | 0.12 | 0.18 | 0.2 | 0.2 | 0.2 | -0 |  |  |  |  |  |  |  |  |  |  |  |  |  |  |  |
| SDQpeer_ct4 | -0.1 | -0.08 | -0 | -0.1 | -0.1 | -0 | 0.5 | -0 |  |  |  |  |  |  |  |  |  |  |  |  |  |  |
| SDQprosocial_ct4 | 0.03 | -0.02 | 0.06 | 0.1 | 0.1 | 0.1 | -0 | 0 | -0 |  |  |  |  |  |  |  |  |  |  |  |  |  |
| RCADS_dep_ct4 | -0.1 | -0.03 | -0.1 | -0.1 | -0.1 | -0 | 0.4 | -0 | 0 | -0.1 |  |  |  |  |  |  |  |  |  |  |  |  |
| RCADS_anx_ct4 | -0.1 | -0.03 | -0.1 | 0 | -0.1 | -0 | 0.5 | -0 | 1 | 0.1 | 0.8 |  |  |  |  |  |  |  |  |  |  |  |
| RCADS_dep_ct5 | -0.2 | -0.09 | -0.1 | -0.1 | -0.2 | -0 | 0.3 | -0 | 0 | -0.1 | 0.6 | 0.4 |  |  |  |  |  |  |  |  |  |  |
| RCADS_anx_ct5 | -0.2 | -0.1 | -0.1 | -0.1 | -0.1 | -0 | 0.3 | 0 | 0 | 0.1 | 0.5 | 0.5 | 0.72 |  |  |  |  |  |  |  |  |  |
| RCADS_dep_pt4 | -0.2 | -0.15 | -0.2 | -0.2 | -0.2 | -0 | 0.2 | -0 | 0 | -0.2 | 0.2 | 0.2 | 0.17 | 0.1 |  |  |  |  |  |  |  |  |
| RCADS_anx_pt4 | -0.4 | -0.24 | -0.3 | -0.2 | -0.2 | -0 | 0.3 | -0 | 0 | -0.1 | 0.2 | 0.2 | 0.14 | 0.1 | 0.8 |  |  |  |  |  |  |  |
| RCADS_dep_pt5 | -0.3 | -0.22 | -0.2 | -0.2 | -0.3 | -0 | 0.1 | -0 | 0 | -0.2 | 0.1 | 0 | 0.41 | 0.2 | 0.7 | 0.6 |  |  |  |  |  |  |
| RCADS_anx_pt5 | -0.3 | -0.23 | -0.2 | -0.2 | -0.1 | -0 | 0.1 | -0 | 0 | 0.1 | 0 | 0.1 | 0.34 | 0.4 | 0.5 | 0.8 | 0.7 |  |  |  |  |  |
| Sex | 0.16 | 0.05 | 0.14 | 0.1 | 0 | 0.1 | 0 | 0 | 0 | 0.3 | 0 | 0.2 | 0.14 | 0.2 | -0.1 | -0.1 | -0 | 0.04 |  |  |  |  |
| SES | 0.22 | 0.26 | 0.19 | 0.1 | 0.2 | 0.2 | 0 | 0 | -0 | 0 | -0 | 0 | -0.2 | -0 | -0.2 | -0.2 | -0.3 | -0.28 | 0.1 |  |  |  |
| lang | 0.81 | 0.82 | 0.82 | 0.7 | 0.8 | 0.8 | -0 | 0 | -0 | 0.1 | -0 | -0 | -0.1 | -0 | -0.2 | -0.3 | -0.3 | -0.22 | 0.2 | 0.26 |  |  |
| lockdown | 0.23 | 0.24 | 0.24 | 0.2 | 0.2 | 0.3 | 0 | 0 | -0 | 0.1 | -0 | -0 | -0.1 | -0 | -0.2 | -0.2 | -0.1 | -0.2 | 0.1 | 0.21 | 0.24 |  |
| NVIQ_z | 0.45 | 0.47 | 0.52 | 0.4 | 0.4 | 0.4 | -0 | -0 | -0 | 0.1 | -0 | -0 | -0.1 | -0 | -0.2 | -0.2 | -0.2 | -0.2 | 0 | 0.2 | 0.58 | 0.2 |

**Figure S1.**


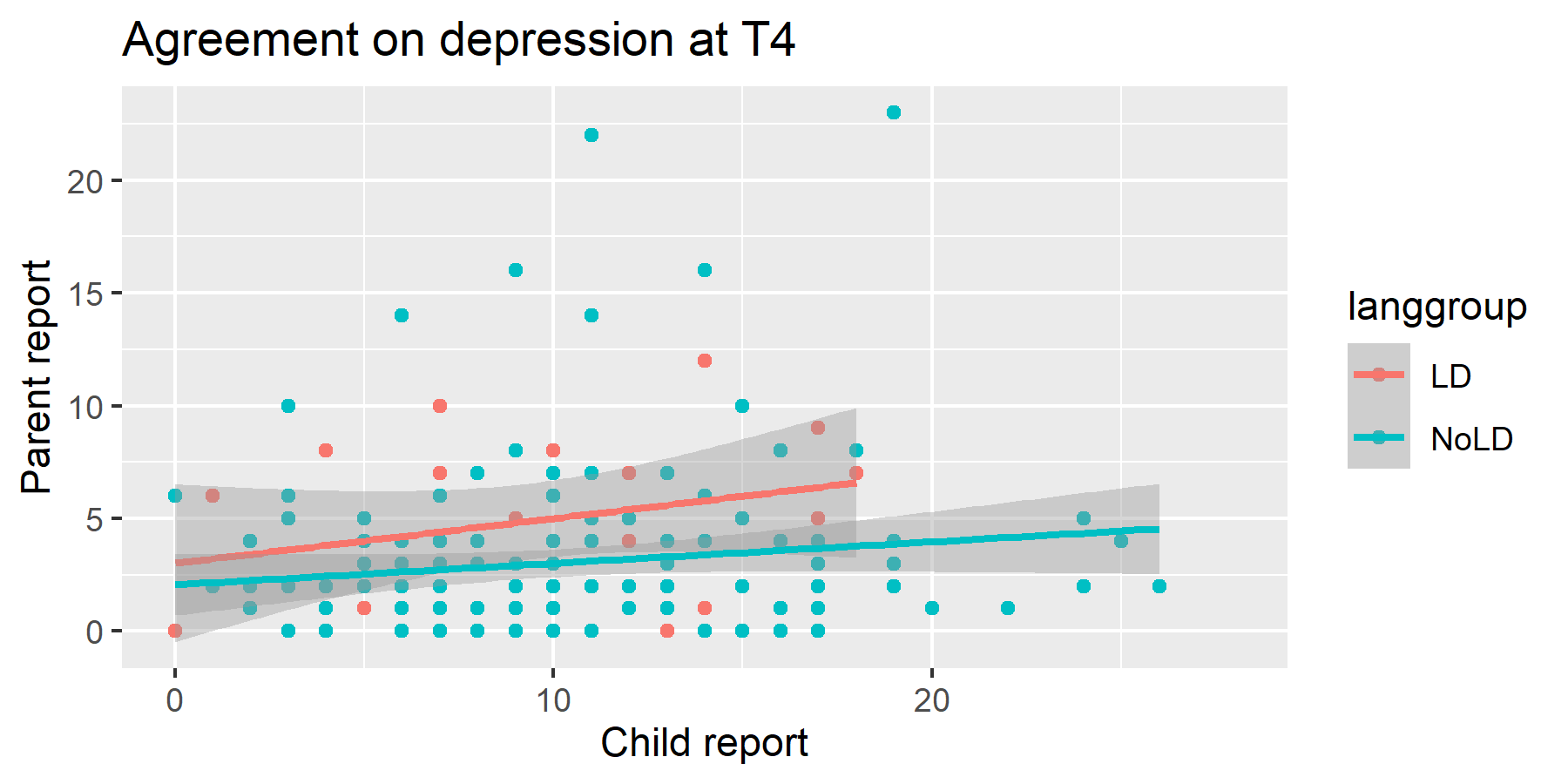
*Correlation between child- and parent-reported depression scores in Year 6*

**Figure S2.**


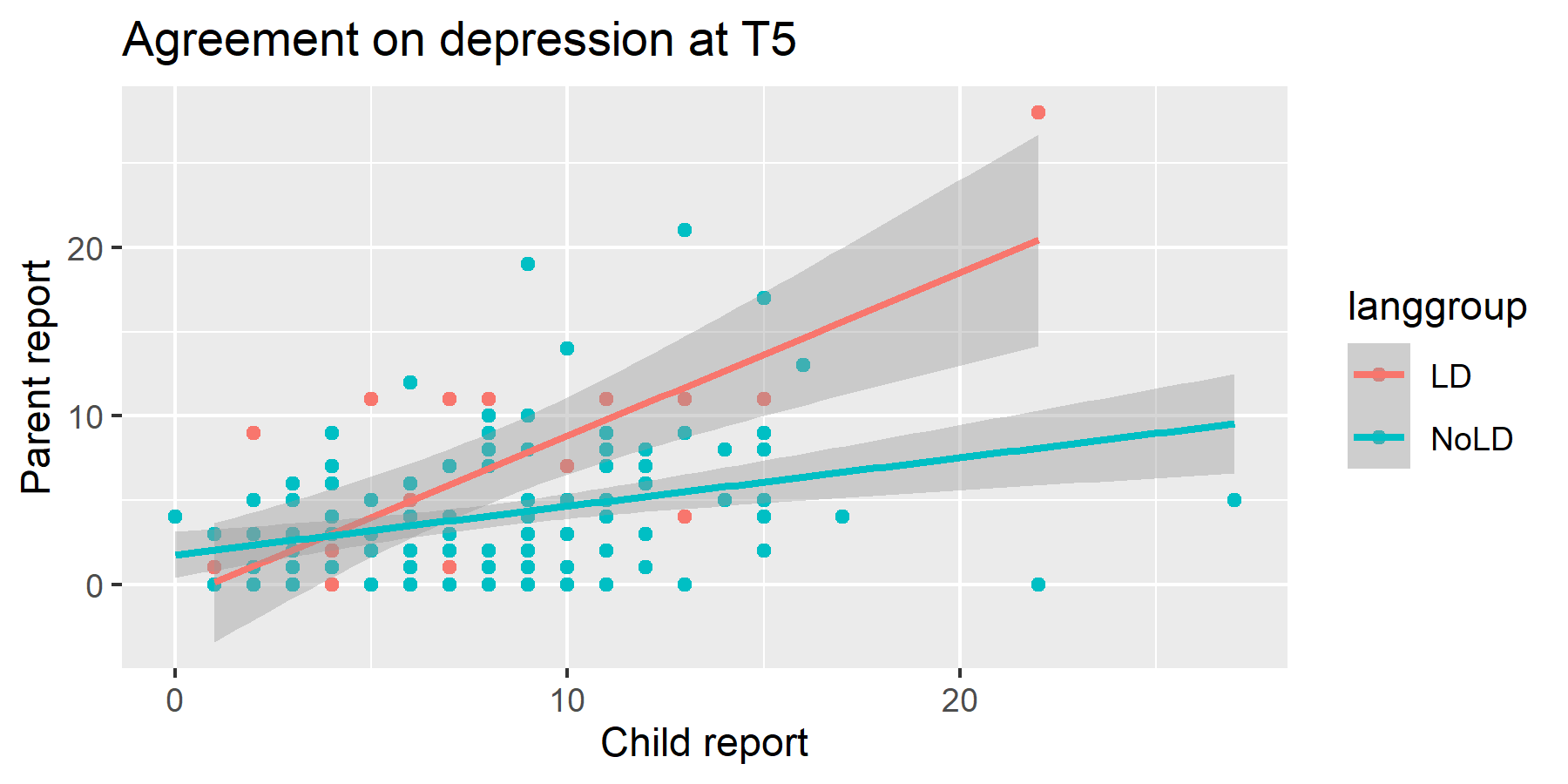
*Correlation between child- and parent-reported depression scores in Year 8*

**Figure S3.**


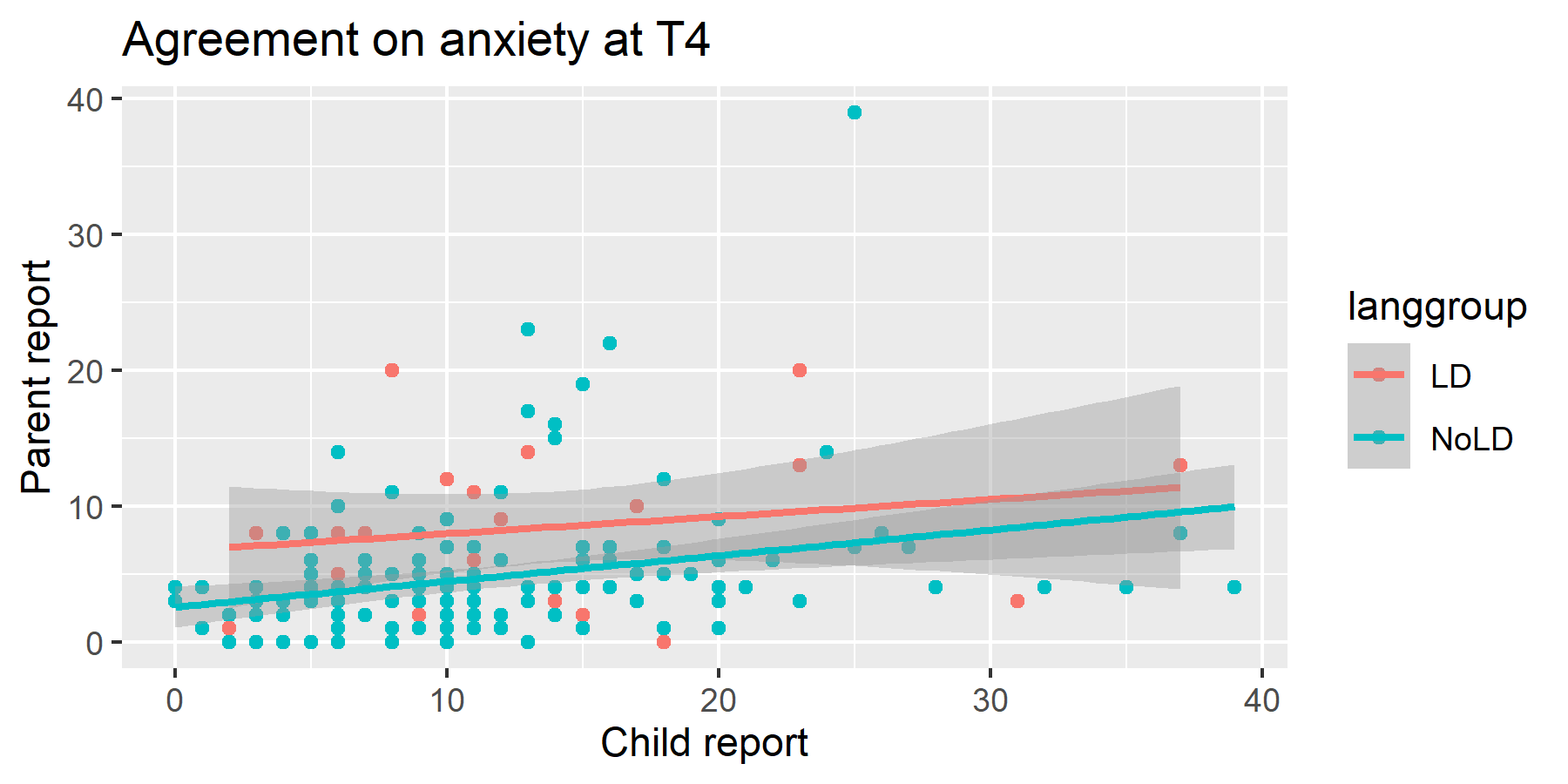
*Correlation between child- and parent-reported anxiety scores in Year 6*

**Figure S4.**

*Correlation between child- and parent-reported anxiety scores in Year 8*


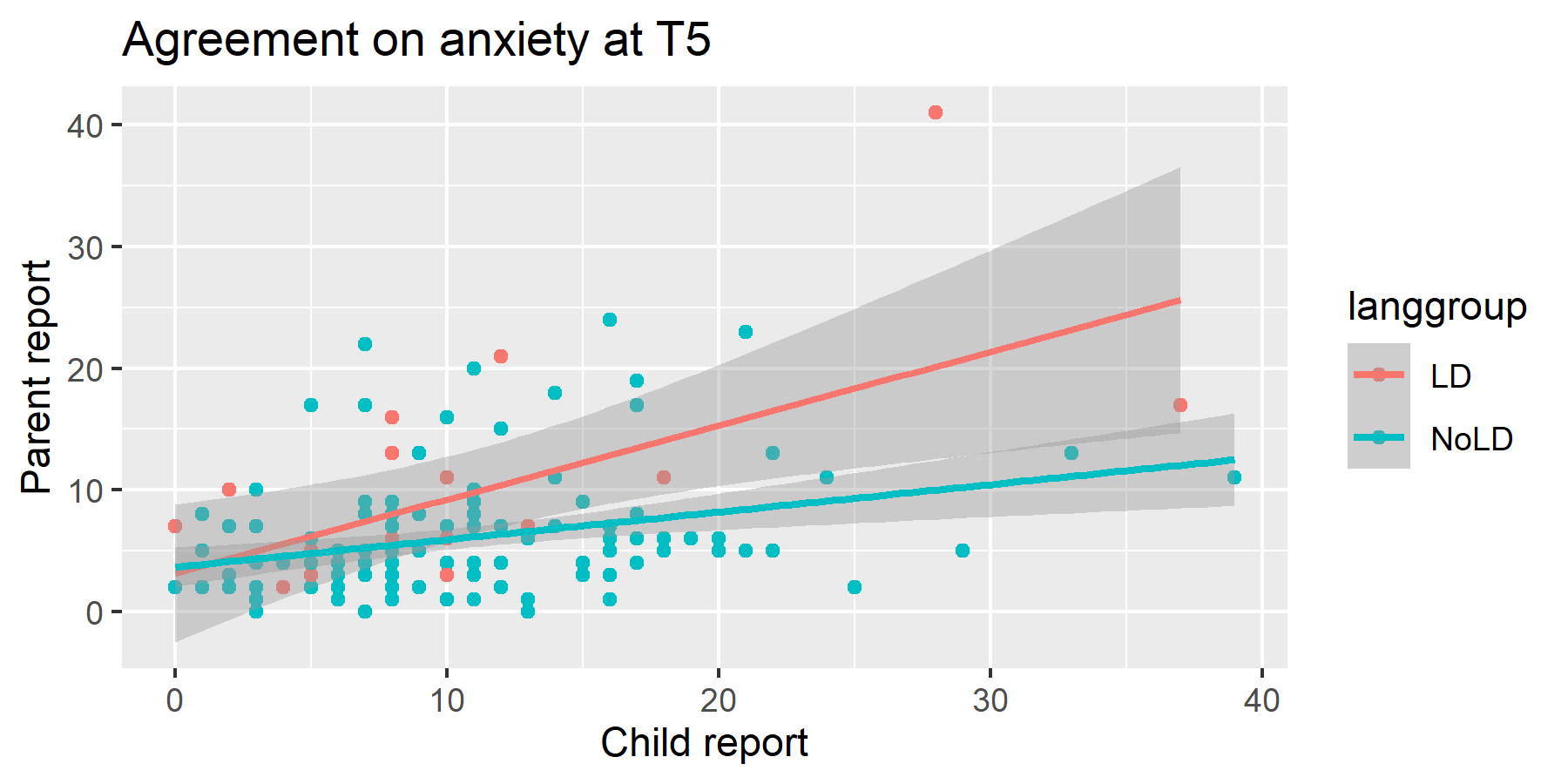

Supplement: sj-docx-1-qjp-10.1177_17470218231158069 – Supplemental material for The role of language in mental health during the transition from primary to secondary education [file sj-docx-1-qjp-10.1177_17470218231158069.docx]
